# The effect of two mineral–vitamin premixes on the blood biochemical parameters, milk yield and composition of Holstein–Friesian cows in Kazakhstan

**DOI:** 10.5194/aab-66-391-2023

**Published:** 2023-12-04

**Authors:** Gulzhan K. Mussayeva, Gulshat I. Shaykamal, Indira N. Aitzhanova, Aigerim Kazhiyakbarova, Jan Miciński, Alicja Sobczak, Nurgul A. Meldebekova, Gulnaz Ilgekbayeva, Nurkuisa M. Rametov

**Affiliations:** 1 Department of Technology Production of Animal Product, Baitursynov Kostanay Regional University, 47 Baytursynov Street, 110000 Kostanay, Republic of Kazakhstan; 2 Department of Sheep and Goat Breeding, University of Warmia and Mazury in Olsztyn, Olsztyn, Poland; 3 Department of Zooengineering, Kazakh National Agrarian Research University, Almaty, Republic of Kazakhstan; 4 Department of GeoSpatial Science Laboratory Institute of Ionosphere, K. I. Satpayev Kazakh National Research Technical University, Almaty, Republic of Kazakhstan

## Abstract

The aim of this study was to determine the effect of two mineral–vitamin premixes on the health status (blood biochemical parameters), milk yield and composition of Holstein–Friesian (HF) cows in Kazakhstan. The study was performed on Holstein–Friesian cows kept on the Bek Plus dairy farm in the village of Korzhynkol, Fyodorovsky District, Kostanay Region. Forty primiparous cows, selected from the herd, were divided by the analogue method into two groups: a control group (C) and an experimental group (E) of 20 animals each. The diets fed to group E cows were supplemented with LI-R 18 PRO and PANTO^®^ Mineral R-77 Premium mineral–vitamin premixes. The premixes positively affected cow productivity and blood biochemical parameters, whose values were higher in group E than in group C. Higher levels of glucose, albumins and globulins exerted a beneficial influence on the health status of cows. No cases of lameness or metabolic diseases were recorded in group E. The yields of milk, protein and fat were higher in group E than in group C. Dietary supplementation with two premixes had no significant effect on the chemical composition of milk, including the values of the following parameters: somatic cell count (SCC), dry matter (DM), lactose ( $L_{\mathrm{se}}$ ), casein ( $C_{\mathrm{in}}$ ), acidity ( $A_{\mathrm{ty}}$ ), lactic acid (LA), density ( $D_{\mathrm{ty}})$ , free fatty acids (FFAs), glucose ( $G_{\mathrm{se}}$ ) and urea ( $U_{\mathrm{ea}}$ ).

## 1 Introduction

The high-yielding Holstein–Friesian (HF) breed is the predominant dairy breed in many countries (Strapakova et al., 2014; Underwood, 1977; Erickson and Kalscheur, 2020), including in Kazakhstan (Bermagambetova et al., 2016). At present, in Kazakhstan, the productivity of HF cows reaches 8000–10 000 kg of milk on breeding farms where cattle are housed in free-stall barns and receive nutritionally balanced diets and 6000–7000 kg of milk on commercial farms (Mironova et al., 2021).

The development of new feed additives can contribute to achieving high milk performance while maintaining the optimal health of dairy herds. Feed additives include premixes that should be characterised by high availability of biologically active substances and high vitamin content, in accordance with the nutrient requirements of high-yielding cows (NRC, 2001). Premixes are often pellet formulations containing B vitamins, optimal doses of choline and biotin and high concentrations of chelated micronutrients (Cu, Zn, Mn and Se).

Good-quality premixes are ideal supplements for dairy cattle rations based on haylage, grass silage and maize silage. Readily available minerals can improve hoof health and prevent ketosis, help balance diets for high-yielding cows, reduce oxidative stress and increase productivity. The ration should be complete and balanced to keep dairy cows healthy and ensure that their blood biochemical parameters remain within reference ranges (Shibata et al., 2015; Shurson et al., 2011).

In northern Kazakhstan, dairy cows are fed mostly preserved feed (silage), especially in winter. However, cows are increasingly being housed indoors all year round, which is why preserved feed should be supplemented with mineral–vitamin premixes. Therefore, efforts are being made to include readily available mineral blends in premixes.

Zinc (Zn), copper (Cu), cobalt (Co), iodine (I) and magnesium (Mg) are essential micronutrients for dairy cows raised in northern Kazakhstan. These trace elements are deficient in this region, but they are required for proper functioning of organs and tissues. The above micronutrients are involved in haematopoiesis and exert protective effects on the body. As a result, they positively affect the productive and reproductive characteristics of cows as well as the growth and development of young cattle (Erickson and Kalscheur, 2020).

Previous studies have shown that the high genetic potential of HF cows for milk production and inadequate feeding, especially during the peripartum period, lead to their premature culling (Sasaki et al., 2017; Weller and Ezra, 2015). High-yielding cows should be analysed during six or more lactation cycles to develop a database of traits that affect their productive lifespan. A knowledge of longevity-promoting traits would support selection of not only heifers, but also cows, so as to maintain a high number of potentially high-yielding cows in the herd. The reasons for a decrease in the productive lifespan of dairy cows include a negative energy balance and a trace mineral deficiency during early lactation when the amount of energy supplied with feed does not meet the energy requirements for milk production. As a result, energy deficiencies are covered by the body's reserves, leading to a decrease in live weight, loss of adipose tissue and metabolic disorders (Butler and Smith, 1989; Cherpanov et al., 2012).

Protein–mineral–vitamin supplements contribute to regulating metabolic processes in dairy cows, thus preventing undesirable metabolic and hormonal changes. Trace mineral deficiencies can lead to a variety of health problems in dairy cows. Essential trace elements play important biological roles and are involved in metabolic processes and physiological functions. They are part of the structures of enzymes, hormones, vitamins and other biologically active compounds. They participate in many biochemical processes by regulating the metabolism of proteins, fats and carbohydrates (Strapakova et al., 2014; Weller and Ezra, 2015).

The aim of this study was to determine the effect of two mineral–vitamin premixes, LI-R 18 PRO and PANTO^®^ Mineral R-77 Premium, on the health status (blood biochemical parameters), milk yield and composition of Holstein–Friesian cows in Kazakhstan.

## 2 Materials and methods

This study was conducted with the approval of the Ethics Committee of the RSE National Center for Biotechnology of the Ministry of Education and Science, RK, Astana, on 25 September 2017. The experiment was performed on 40 primiparous HF cows kept in a herd of 900 cows on the Bek Plus dairy farm in the village of Korzhynkol, Fyodorovsky District, Kostanay Region. The cows were divided into two groups: a control group (C) and an experimental group (E) of 20 animals each, analogous in terms of body weight (BW) (approximately 550–570 kg on average), age at first calving (24–28 months) and daily milk yield within the first 5 to 10 d post-calving (above 25 kg).

In order to achieve the main aim of the study, the following specific research objectives were formulated: to establish the optimal dose of a supplement consisting of two premixes, to analyse the blood morphological and biochemical parameters of cows, and to determine milk yield and composition.

**Table 1:** Average daily feed intake by primiparous cows in the control and experimental groups from calving to day 60 of lactation.

Feed (kg)	Average daily	
	feed supply in	
	the control and	
	experimental	
	groups (kg)	
	C	E
Hay	1.00	
Maize silage	25.00	
Alfalfa haylage	13.00	
Ground oat and barley grain	2.50 + 3.00	
Sunflower meal	4.00	
Salt lick (NaCl)	0.130	
LI-R 18 PRO premix	–	0.100
PANTO^®^ Mineral R-77 Premium premix	–	0.100

Cows in both groups received identical diets formulated in accordance with NRC recommendations (NRC, 2001). The main roughages were hay, maize silage and haylage. Apart from roughage, the daily ration also contained 750 to 900 g of concentrate per 100 kg BW. Such a ration was sufficient to achieve an average daily milk yield exceeding 20 kg for 305 d lactation. In group E, the ration was supplemented with two mineral–vitamin premixes: LI-R 18 PRO and PANTO^®^ Mineral R-77 Premium, at 100 g of each product. Group C animals did not receive any mineral–vitamin supplements (Table 1).

The nutritional values of the diets and differences in the nutrient levels are shown in Table 2.

**Table 2:** Nutritional values of feed rations for primiparous cows according to NRC (2001).

Nutrient	Group		Differences betweengroup E compared
			to group C (%)
	C	E	
Dry matter (g)	20 200	21 086	+4.30
Crude protein (g)	3012	3072	+1.7
Digestible protein (g)	2060	2156	+4.5
Lysine (g)	142	148	+4.2
Methionine (g)	71	74	+7.3
Tryptophan (g)	51	50	-2.0
Crude fibre (g)	4160	4987	+26.6
Starch (g)	3160	2959	-6.4
Sugar (g)	1960	398	-4.9
Crude fat (g)	820	928	+21.7
NaCl (g)	130	130	Identical
Ca (g)	130	128.60	-1.1
P (g)	92	86.90	-5.6
Fe (mg)	1522	2259	+48.4
Cu (mg)	187	168	-10.1
Zn (mg)	1235	1176	-4.8
Co (mg)	15.0	14	-5.4
Mn (mg)	1137	908	-25.2
J (mg)	17.6	15	-10.3
β -Carotene (mg)	820	709	-13.6
Vitamin A – retinol (mg)	328 000	383 540	+16.9
Vitamin D3 – cholecalciferol (mg)	184 500	243 800	+32.1
Vitamin E – tocopherol (mg)	780	802	+2.8
Energy NEL per kilogram per DM (MJ)	1.40	1.30	-7.15

In northern Kazakhstan, the LI-R 18 PRO mineral–vitamin premix is used as a feed additive to normalise and maintain a healthy metabolism in dairy cows with a daily milk yield of 25 kg. The recommended daily dose of this product is 100 g per cow. The premix is composed of mono-calcium phosphate, Zn, yeasts (*Saccharomyces cerevisiae*) and high concentrations of vitamins A, B, D3 and E. Mono-calcium phosphate ensures the highest bioavailability of phosphorus (P) and calcium (Ca). Zinc contributes to the health and integrity of hooves, prevents parakeratosis and reduces the incidence of lameness in cows. Live yeasts stabilise ruminal pH, which increases feed intake, stimulates microbial protein synthesis, reduces the somatic cell count (SCC) in milk and improves reproductive efficiency. In addition, B vitamins contribute to stabilising ruminal microbiota and improving feed fermentation. The premix is easy to dose and has a pleasant taste and smell, which increases the intake of the basal diet.

PANTO^®^ Mineral R-77 Premium is another mineral–vitamin premix tested in this study. Its recommended daily dose is also 100 g per cow. The premix enriches the basal diet with minerals such as selenium (Se), Zn, Ca, P and Mg. High-yielding cows have a high metabolic rate, and many biologically active compounds are released with milk. Therefore, dairy cows have high dietary mineral and vitamin requirements. This applies in particular to primiparous cows, whose colostrum is usually of a lower quality than that of multiparous cows (Zwierzchowski et al., 2020). Premixes should be administered to dairy cows fed high-concentrate diets that increase the risk of metabolic disorders such as acidosis and ketosis.

It is recommended to combine both analysed premixes at a daily dose of 200 g per cow (100 g of each product) to diversify the mineral–vitamin blend and to enhance its health effects. The feed ration supplemented with both premixes is richer in biologically active substances and minerals since the pellets contain B vitamins, choline and biotin as well as high levels of chelated micronutrients, i.e. Cu, Zn, Mn and Se. The composition of both premixes is shown in Table 3.

**Table 3:** Composition of premixes in 1 kg.

Composition	PANTO^®^	LI-R 18 PRO
	Mineral R-77	
	Premium	
Calcium (%)	23.0	18.0
Phosphorus (%)	3.0	6.0
Sodium (%)	7.0	9.0
Magnesium (%)	6.0	4.0
Vitamin A (IU)	500 000	1 000 000
Vitamin D3 (IU)	150 000	100 000
Vitamin E (mg)	2500	2000
Vitamin B1 – thiamine (mg)	–	20
Vitamin B2 (mg)	–	18
Vitamin B6 (mg)	–	10
Vitamin K (mg)	–	5
Nicotinic acid (mg)	–	110
Pantothenic acid (mg)	–	70
Folic acid ( µ g)	–	10 000
Niacin (mg)	20 000	–
Biotin ( µ g)	50 000	–
Choline (mg)	15 000	–
Copper (mg)	450	700
Zinc (mg)	3500	9000
Manganese (mg)	2150	3000
Iodine (mg)	24	100
Cobalt (mg)	16	20
Selenium (mg)	25	40
*Saccharomyces cerevisiae* (mg)	–	26 400

*Saccharomyces cerevisiae* – MUCL 39885 – $15\times 10^{9}$ KBE g ${}^{-1}$ .

The yields of milk, protein and fat (kg) were determined for full and 305 d lactations. The following indicators of productive and reproductive cycles (days) were analysed: lactation length, inter-pregnancy interval, inter-calving interval, length of the dry period, length of the gestation period as well as the insemination index (number of insemination doses per conception).

Milk samples for qualitative composition analyses were collected from group C and E cows in each month of lactation. The analyses were performed in the laboratory of Baitursynov Kostanay State University. The milk samples were collected and prepared for analysis according to the methodology of GOST, 13928-84 (1984) and GOST RK, 52738 (2007). The following physiochemical parameters of milk were analysed: SCC (1000 cm ${}^{-3}$ ), fat – $F_{\mathrm{tt}}$ (% and kg), protein – $P_{\mathrm{in}}$ (% and kg), dry matter – DM (% and kg), lactose – $L_{\mathrm{se}}$ (% and kg), casein – $C_{\mathrm{in}}$ (% and kg), lactic acid – LA (%), free fatty acids – FFAs ( µ mol L ${}^{-1}$ ), glucose – $G_{\mathrm{se}}$ (g per 100 mL), urea – $U_{\mathrm{ea}}$ (mmol L ${}^{-1}$ ), density – $D_{\mathrm{ty}}$ (kg m ${}^{-3}$ ) and acidity – $A_{\mathrm{ty}}$ ( ${}^{o}$ SH: Henkel–Soxhlet degrees). The physicochemical properties of the milk and SCC were determined with the use of MilkoScan FT-1 (Foss, Denmark) and Ecomilk AMB-1-03 (Bulteh 2000, Bulgaria) analysers.

Blood samples (10 mL) for biochemical analyses were collected from the jugular vein of each cow before morning milking and feeding with the use of vacutainer tubes for collecting whole blood samples. The following blood biochemical parameters were determined: total protein (g L ${}^{-1}$ ), glucose ( µ mol L ${}^{-1}$ ), cholesterol (mmol L ${}^{-1}$ ), alkaline phosphatase (U L ${}^{-1}$ ), urea (mmol L ${}^{-1}$ ), FFAs (mmol L ${}^{-1}$ ), albumins (g L ${}^{-1}$ ), globulins (g L ${}^{-1}$ ), β -carotene (mg dL ${}^{-1}$ ), bilirubin ( µ mol L ${}^{-1}$ ), Ca (mmol L ${}^{-1}$ ), P (mmol L ${}^{-1}$ ) and Mg (mmol L ${}^{-1}$ ), with the use of an XT-2000i automated hematology analyser (Sysmex). Blood glucose concentration was determined using an A15 automated biochemistry analyser (Biosystems).

The results were processed statistically using Statistica version 13 software (StatSoft Inc., 2023). The significance of differences between mean values in groups was determined by a Student's t test at p≤0.01 and p≤0.05 .

## 3 Results

In general, the nutritional value of the diets was consistent with NRC standards (NRC, 2001), and they were balanced so as to meet the nutrient requirements of cows. An excess of crude fibre in diet E did not compromise the health status, gastrointestinal function or productivity of the cows. Also, a higher crude fat content did not have a negative effect on the experimental cows.

The blood biochemical parameters are presented in Table 4 and are compared with the reference ranges reported in the literature.

**Table 4:** Blood biochemical parameters of primiparous cows.

Specification	Group C	Group E	Reference	References
	LSM ± SD	LSM ± SD	range	
Total protein (g L ${}^{-1}$ )	68.88 ${}^{b}$ ± 1.04	71.00 ${}^{a}$ ± 0.54	51–71	Winnicka (2004)
Glucose ( µ mol L ${}^{-1}$ )	2.90 ${}^{b}$ ± 0.07	3.10 ${}^{a}$ ± 0.08	>3.0	Whitaker et al. (2005)
Cholesterol (mmol L ${}^{-1}$ )	2.68 ${}^{B}$ ± 0.32	4.20 ${}^{A}$ ± 0.14	3.98–9.93	Kuleta (2005)
Alkaline phosphatase (U L ${}^{-1}$ )	53.93 ${}^{a}$ ± 1.60	42.30 ${}^{b}$ ± 3.57	up to 200	Baumgartner (2005)
Urea (mmol L ${}^{-1}$ )	2.64 ${}^{b}$ ± 0.26	3.38 ${}^{a}$ ± 0.13	3.5–5.0	Baumgartner (2005)
Free fatty acids (mmol L ${}^{-1}$ )	0.54 ${}^{b}$ ± 0.12	0.70 ${}^{a}$ ± 0.20	0.5–0.7	Whitaker et al. (2005)
Ca (mmol L ${}^{-1}$ )	2.36 ${}^{a}$ ± 0.02	2.07 ${}^{b}$ ± 0.04	2.0–2.5	Winnicka (2004)
P (mmol L ${}^{-1}$ )	1.90 ± 0.02	1.66 ± 0.11	1.6–2.3	Baumgartner (2005)
Albumins (g L ${}^{-1}$ )	34.97 ${}^{b}$ ± 0.47	37.73 ${}^{a}$ ± 1.10	>30	Whitaker et al. (2005)
Globulins (g L ${}^{-1}$ )	33.81 ${}^{b}$ ± 1.64	38.27 ${}^{a}$ ± 0.75	<50	Whitaker et al. (2005)
Mg (mmol L ${}^{-1}$ )	0.93 ${}^{b}$ ± 0.02	1.11 ${}^{a}$ ± 0.02	>0.7	Whitaker et al. (2005)
β -Carotene (mg dL ${}^{-1}$ )	0.19 ${}^{b}$ ± 0.02	0.34 ${}^{a}$ ± 0.01	<5	Baumgartner (2005)
Bilirubin ( µ mol L ${}^{-1}$ )	4.55 ${}^{b}$ ± 0.35	5.02 ${}^{a}$ ± 0.18	1.9–7.0	Winnicka (2004)

Significant differences at A and B are p≤0.01 , and at a and b they are p≤0.05 . SD: standard deviation.

Group E cows, compared with group C animals, were characterised by significantly ( p ≤ 0.01 and p ≤ 0.05) higher blood levels of total protein (71 g L ${}^{-1}$ ), glucose (3.10 µ mol L ${}^{-1}$ ), cholesterol (4.20 mmol L ${}^{-1}$ ), urea (3.38 mmol L ${}^{-1}$ ), FFAs (0.70 mmol L ${}^{-1}$ ), albumins (37.73 g L ${}^{-1}$ ), globulins (38.27 g L ${}^{-1}$ ), Mg (1.11 mmol L ${}^{-1}$ ), β -carotene (0.34 mg dL ${}^{-1}$ ) and bilirubin (5.02 µ mol L ${}^{-1}$ ) and by significantly lower blood levels of acid phosphatase (42.30 U L ${}^{-1}$ ), Ca (2.07 mmol L ${}^{-1}$ ) and P (1.66 mmol L ${}^{-1}$ ). It should be stressed that all the analysed blood parameters remained within the reference ranges for dairy cows (Table 4). In comparison with group C, higher levels of total protein, glucose, albumins and globulins in the blood of group E cows positively affected their overall health status.

The average daily milk yield calculated for full and 305 d (standard) lactations and converted to fat-corrected milk (FCM) content was higher in group E. Milk from group E cows had a lower fat content (by 0.16 %) than milk from group C cows, and therefore the FCM yield was lower in group E. In turn, the average protein content of milk was higher in group E. In both groups, the SCC was below the maximum allowable level of 400 000 cells mL ${}^{-1}$ , which points to the high quality of the analysed milk (Table 5).

**Table 5:** Milk yield of primiparous cows (LSM ± SD).

Specification	Group	
	C	E
Cows (head)	20	20
Milk yield for 305 d lactation (kg d ${}^{-1}$ )	23.5 ${}^{b}$ ± 4.14	28.4 ${}^{a}$ ± 4.12
Milk yield for full lactation (kg d ${}^{-1}$ )	19.3 ${}^{b}$ ± 4.14	22.9 ${}^{a}$ ± 4.12
Fat (%)	3.94 ${}^{a}$ ± 0.25	3.78 ${}^{b}$ ± 0.31
Protein (%)	3.11 ${}^{b}$ ± 0.08	3.25 ${}^{a}$ ± 0.12
Somatic cell count ( ×1000 cells mL ${}^{-1}$ )	94.51 ${}^{a}$ ± 43.48	83.23 ${}^{b}$ ± 41.78

Significant differences at A and B are p ≤ 0.01, and at a and b they are p ≤ 0.05.

An analysis of the milk performance and indicators of productive and reproductive cycles in primiparous cows revealed that lactation, inter-calving interval and inter-pregnancy interval were significantly ( p ≤ 0.01) shorter in group E than in group C (by 35, 46 and 49 d, respectively) (Table 5).

**Table 6:** Indicators of the productive and reproductive performances of primiparous cows (LSM ± SD).

Specification	Group		Average
	C	E	
Lactation length (d)	449 ${}^{A}$ ± 21.34	414 ${}^{B}$ ± 16.69	432 ± 19.02
Length of the dry period (d)	41 ± 2.81	40 ± 5.92	41 ± 4.37
Inter-calving interval (d)	490 ${}^{A}$ ± 27.55	444 ${}^{B}$ ± 32.07	467 ± 29.81
Inter-pregnancy interval (d)	207 ${}^{A}$ ± 12.88	158 ${}^{B}$ ± 14.31	183 ± 13.60
Insemination index (number of insemination doses per conception)	1.5 ± 0.22	1.3 ± 0.36	1.4 ± 0.29
Milk yield for full lactation (kg)	8665 ${}^{B}$ ± 231.03	9480 ${}^{A}$ ± 350.77	9073 ± 290.92
Milk yield for 305 d lactation (kg)	7167 ${}^{B}$ ± 154.28	8652 ${}^{A}$ ± 222.14	7910 ± 188.21
${}^{*}$ FCM = (0.4 ⋅ M + 15 ⋅ F ) (kg d ${}^{-1}$ )	8581 ${}^{B}$ ± 142.26	9162 ${}^{A}$ ± 94.37	8872 ± 132.03
Fat yield for full lactation (kg)	341 ${}^{b}$ ± 21.34	358 ${}^{a}$ ± 18.39	353 ± 19.87
Protein yield for full lactation (kg)	269 ${}^{b}$ ± 16.71	308 ${}^{a}$ ± 15.38	289 ± 16.05
Fat content (%)	3.94 ${}^{a}$ ± 0.89	3.78 ${}^{b}$ ± 0.92	3.89 ± 0.91
Protein content (%)	3.11 ${}^{b}$ ± 0.12	3.25 ${}^{a}$ ± 0.13	3.18 ± 0.12

${}^{*}$ FCM (fat-corrected milk) is milk with a standard fat content. Significant differences at A and B are p ≤ 0.01, and at a and b they are p ≤ 0.05.

In the first reproductive cycle, the insemination index was lower in group E, pointing to higher conception rates in this group. Milk fat and protein yields were higher in group E, whereas the milk fat content was lower in group E compared with group C. In turn, the milk protein content was higher ( p ≤ 0.05) in group E (Table 6).

**Figure 1:**
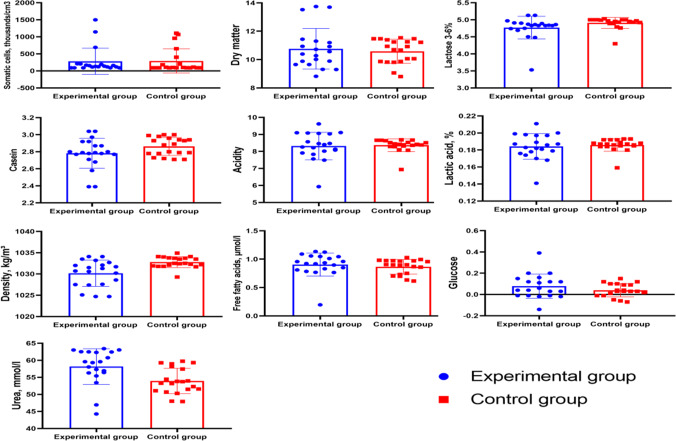
Chemical, physical and quality features of milk.

Dietary supplementation with two premixes had no significant effect on the chemical composition of milk, including the values of the following parameters: SCC, DM, $L_{\mathrm{se}}$ , $C_{\mathrm{in}}$ , $A_{\mathrm{ty}}$ , LA, $D_{\mathrm{ty}}$ , FFAs, $G_{\mathrm{se}}$ and $U_{\mathrm{ea}}$ (Fig. 1).

## 4 Discussion

Milk performance is the key indicator of the productive efficiency of dairy cattle breeds. Milk performance parameters (milk yield, milk fat and protein content) are determined by an animal's genotype and environmental factors (Mikhaylovich et al., 2018).

Maintaining welfare standards and feeding high-quality diets that meet the nutrient requirements of animals at different physiological stages are important considerations in Holstein cattle breeding (Mironova et al., 2021). A key role is played by rational nutrition and feeding a complete balanced ration, which enable dairy cattle to realise their full genetic potential and improve productivity. In addition to organic and bioactive compounds, balanced diets for lactating cows should also contain minerals, including macro-minerals and micro-minerals, which are often found to be inadequate in forage. Therefore, it is common practice on dairy farms to supplement cow diets with minerals (Erickson and Kalscheur, 2020).

According to Martens and Bange (2013), many cows are already culled after two to three calvings, i.e. before reaching maximum milk production capacity. Djedović et al. (2021) demonstrated that high-yielding cows that have given birth to five to seven calves are particularly valuable for breeding herds because only animals characterised by a strong build and desirable conformation traits are able to produce large quantities of milk for many years and are resistant to various diseases. They are also included in progeny testing programmes and can be identified as potential bulls and dams of high genetic merit and bred to produce young bulls or even to become ancestors of valuable families.

According to Vanegas et al. (2004), adequate levels of Ca and P in the ration exert positive effects on a wide range of body functions in dairy cows, bone tissue and hoof health. Phosphorus contributes to dietary crude protein assimilability by stimulating microbial growth in the ruminal forestomach (Suttle, 2010). Zinc, which is involved in keratin production, is also important for hoof health (Malestein, 1995). Zinc deficiency may manifest as interdigital dermatitis (Hill and Shannon, 2019). It should be stressed that, in the present study, no cases of lameness were noted in group E, whereas three such cases were recorded in group C.

An excess of crude fat in diet E ( + 13.17 %) had no negative effect on the experimental cows. According to the literature, a dietary fat content of 4.2 % (DM basis) has a beneficial influence on rumen function and stimulates microbial protein synthesis (Underwood, 1977).

Significant changes in blood biochemical parameters may be observed in early lactation. According to Mordak and Nicpoń (2006), a minor increase in the total bilirubin concentration in cows following parturition is a normal physiological phenomenon resulting from an increase in “indirect” bilirubin concentrations due to red blood cell rupture (haemolytic jaundice). In this period, the activity of aspartate transaminase (AST) may increase and the concentrations of glucose, potassium, chlorides, Ca and inorganic P may decrease. Hypoglycaemia can also develop in the early postpartum period, pointing to serious health problems in cows. Hypoglycaemia is diagnosed when blood glucose levels drop below 3.9 mmol L ${}^{-1}$ (Mordak, 2008). In the present study, blood glucose levels remained within the reference range in both groups, but higher values were noted in group E. None of the cows showed apparent symptoms of hypoglycaemia or muscular dystrophy, which also testifies to normal blood glucose levels.

In the current experiment, the blood levels of bilirubin (the product of haemoglobin breakdown) were higher in group E than in group C, but they remained within the reference range. Therefore, bilirubin exerted no toxic effects on cows, most likely because excess bilirubin was neutralised by albumin, thus preventing bilirubin toxicity (Preś and Mordak, 2010). Mirowski (2019) found that mineral–vitamin supplements administered to cows helped reverse nutritional deficiencies and bring back and maintain normal levels of micronutrients.

In the present study, HF cows were characterised by normal blood concentrations of albumins and globulins. This suggests that a properly functioning immune system effectively protected high-yielding cows in group E during intensive milk production. Blood β -carotene levels were low in group C and much higher in group E. It can be assumed that vitamin A contained in the mineral–vitamin premix compensated for the β -carotene deficiency in group E.

Numerous studies have shown that minerals and vitamins added to dairy cattle rations contribute to a significant increase in milk production in the first 120 d of lactation. In a study by Strusińska et al. (2006), a mineral–vitamin premix given to dairy cows increased the yields of protein (by 4.7 %), fat (by 4.8 %) and DM (by 2 %) relative to the control group. Czaplicka et al. (2014) reported that the addition of live yeast cultures (*Saccharomyces cerevisiae*) to diets for dairy cows increased their daily milk yield by 3.56 kg, while Iwańska et al. (1999) reported that cows supplied with the yeast culture produced 38 kg more milk than the controls by day 120 of lactation, and from cows fed yeast culture and premix an additional 150 kg of milk was obtained. Skórko-Sajko et al. (1993) also confirmed that yeast and mineral supplements exerted a highly significant impact on milk production. Other authors also observed a positive influence of mineral supplements on the milk performance of dairy cows (Bielak, 1995; Kinal et al., 2007; Wu and Satter, 2000). Matras et al. (2005) evaluated the efficacy of mineral mixtures balancing the mineral nutrition of dairy cows in eight selected barns using different feeding models and found that the mineral mixture used in barns with a traditional feeding model increased milk yield by 8 % and significantly ( p≤0.05 ) increased the levels of deficient minerals (Ca, P, K and Cu) in the blood serum of cows. According to Krzyżewski et al. (2014), in the light of current knowledge, milk production is determined by the energy density of diets in 50 %, protein concentration in 30 % and the levels of available minerals and vitamins in 20 %. Van Emon et al. (2020) reported that trace minerals considerably affected the health status and milk performance of dairy cows due to alterations in enzymatic function, DNA replication and the formation of antioxidants.

## 5 Conclusions

Two premixes added to diets for group E cows positively affected blood biochemical parameters whose values were higher in group E than in group C. Higher levels of glucose, albumins and globulins exerted a beneficial influence on the health status of cows. No cases of lameness or metabolic diseases were recorded in group E. The yields of milk, protein and fat were higher in group E than in group C. Dietary supplementation with two premixes had no significant effect on the chemical composition of milk, including the values of the following parameters: SCC, DM, $L_{\mathrm{se}}$ , $C_{\mathrm{in}}$ , $A_{\mathrm{ty}}$ , LA, $D_{\mathrm{ty}}$ , FFAs, $G_{\mathrm{se}}$ and $U_{\mathrm{ea}}$ .

## Data Availability

The original data of the paper are available from the corresponding author upon reasonable request.
